# Differentiating stages of functional vision loss from glaucoma using the Disc Damage Likelihood Scale and cup:disc ratio

**DOI:** 10.1136/bjo-2022-321643

**Published:** 2023-01-18

**Authors:** Heiko Philippin, Einoti Naino Matayan, Karin Marianne Knoll, Edith Macha, Sia Mbishi, Andrew Makupa, Cristóvão Daniel Matsinhe, Isac Vasco da Gama, Mário Jorge Monjane, Joyce Awum Ncheda, Francisco Alcides Francisco Mulobuana, Elisante Muna, Nelly Fopoussi Guylene, Gus Gazzard, Ana Patricia Marques, Peter Shah, David Macleod, William Makupa, Matthew J Burton

**Affiliations:** 1 International Centre for Eye Health, London School of Hygiene and Tropical Medicine Department of Clinical Research, London, UK; 2 Eye Centre, Medical Centre, Faculty of Medicine, University of Freiburg, Freiburg im Breisgau, Germany; 3 Eye Department, Kilimanjaro Christian Medical Centre, Moshi, Tanzania, United Republic of; 4 Kilimanjaro Christian Medical University College, Moshi, Tanzania, United Republic of; 5 Provincial Hospital of Pemba, Pemba, Mozambique; 6 Hospital Central de Quelimane, Quelimane, Mozambique; 7 Presbyterian Eye Hospital, Bafoussam, Cameroon; 8 Eye Department, Cameroon Baptist Convention Health Services, Douala, Cameroon; 9 National Institute for Health Research (NIHR) Biomedical Research Centre (BRC) for Ophthalmology at Moorfields Eye Hospital NHS Foundation Trust and UCL Institute of Ophthalmology, London, UK; 10 Institute of Ophthalmology, University College London, London, UK; 11 Ophthalmology, University Hospitals Birmingham NHS Foundation Trust, Birmingham, UK; 12 MRC International Statistics & Epidemiology Group, London School of Hygiene & Tropical Medicine, London, UK

**Keywords:** Glaucoma, Diagnostic tests/Investigation

## Abstract

**Background:**

Glaucoma staging is critical for treatment planning but has rarely been tested in severe/end-stage disease. We compared the performance of the Disc Damage Likelihood Scale (DDLS) and cup:disc ratio (CDR) using a functional glaucoma staging system (GSS) as the reference standard.

**Methods:**

Post hoc analysis of a randomised controlled trial at the Eye Department of Kilimanjaro Christian Medical Centre, Tanzania. Eligible participants (aged ≥18 years) with open-angle glaucoma, intraocular pressure (IOP) of >21 mm Hg, were randomised to timolol 0.5% eye drops or selective laser trabeculoplasty. Fundoscopy established vertical and horizontal CDRs and DDLS. Visual acuity and static visual fields were graded (GSS). The study used area under the receiver operating characteristic (AROC) curves and Spearman’s rank correlation coefficients to compare staging systems. Logistic regression with generalised estimating equations determined risk factors of functional severe/end-stage glaucoma.

**Results:**

382 eyes (201 participants) were evaluated; 195 (51%) had severe or end-stage glaucoma; mean IOP was 26.7 (SD 6.9) mm Hg. DDLS yielded an AROC of 0.90 (95% CI 0.87 to 0.93), vertical cup:disc ratio (vCDR) of 0.88 (95% CI 0.85 to 0.91, p=0.048) for identifying severe/end-stage disease. Correlation coefficients comparing GSS to DDLS and vCDRs were 0.73 and 0.71, respectively. Advanced structural stages, vision impairment, higher IOP and less financial resources were risk factors of functional severe/end-stage glaucoma.

**Conclusion:**

This study indicates that both structural staging systems can differentiate severe/end-stage glaucoma from less severe disease, with a moderate advantage of DDLS over CDR. Clinical examination of the optic disc plays an important role in addition to functional assessment when managing severe/end-stage glaucoma.

**WHAT IS ALREADY KNOWN ON THIS TOPIC**

- Functional and structural descriptors of the optic nerve head damage can be used to distinguish between different stages of glaucoma, with most diagnostic studies focusing on earlier stages.
- We assessed eyes with predominantly later stages of glaucoma

**WHAT THIS STUDY ADDS**

- Disc Damage Likelihood Scale and cup:disc ratio are feasible methods to discriminate late functional stages of glaucoma.

**HOW THIS STUDY MIGHT AFFECT RESEARCH, PRACTICE OR POLICY**

- These low-cost structural grading systems can support treatment planning for late stage glaucoma, which has a particularly negative impact on visual function and quality of life.

## Introduction

Glaucoma is the most common cause of irreversible blindness worldwide, leading to reduced quality of life and livelihood.^1^ Sight loss from glaucoma is a result of damage to ocular nerve fibre tissue, mainly caused by increased intraocular pressure (IOP). Staging the damage is important for monitoring the progression of the disease and planning management accordingly. This typically includes appropriate reduction of IOP, along with other components of glaucoma care. Progression of this glaucomatous nerve fibre damage can be monitored with both functional and anatomical descriptors.

Functional glaucomatous damage is usually measured by static visual field (VF) examination (perimetry), with disease staging based on the extent and severity of field loss.^2^ However, severe and end-stage glaucoma commonly affect the central visual acuity, so that static VF testing cannot be reliably performed due to the eye’s inability to fixate. Under these conditions, visual acuity can be used as an alternative means to describe advanced functional damage. Mills *et al.* proposed a glaucoma staging system (GSS) based on static VF examinations, and added categories for severe and end-stage glaucoma; the latter applies if a static VF test cannot be performed due to a central scotoma or the eye has a visual acuity of ≤20/200.^3^ This provides for categorisation of glaucomatous functional damage ranging from prediagnosis to end-stage disease.

Assessment of anatomical or structural damage due to glaucoma focuses mainly on the optic nerve head rim and cup, formed by optic nerve fibres.^4^ The most commonly used grading system measures the cup:disc ratio (CDR), usually by slit-lamp indirect ophthalmoscopy. Armaly described it in 1967 as the ratio of the vertical and horizontal diameters of the optic disc cup to the overall diameters of the disc.^5^ Spaeth *et al.* later developed the Disc Damage Likelihood Scale (DDLS), which identifies the narrowest rim width in relation to the disc diameter (rim:disc ratio). If no rim is present anymore in a particular sector of the disc, the scale quantifies the circumferential extent of the rim loss.^6^ This allows a structural grading ranging from a normal optic nerve head to a complete loss of the neuroretinal rim in the final stage of the disease.

Many glaucoma diagnostic studies have focused on distinguishing between normal eyes and early or moderate glaucoma typically with preserved central visual acuity, using perimetry as the main method for disease staging. More advanced glaucoma is often associated with a reduced visual acuity which has additional negative effects on mental health status, morbidity, mortality and the cost of glaucoma management.^7^ Each further stage of glaucoma can lead to relevant changes in quality of life.^8 9^ Worldwide, advanced glaucoma is more prevalent in low-resource settings where expensive equipment might be less available.^10–12^ Our aim was to evaluate the low-cost structural DDLS and CDR grading systems for their ability to discriminate different functional stages of glaucoma in a study population with predominantly advanced disease.

## Methods

### Study design

This study was based on a post hoc analysis of the Kilimanjaro Glaucoma Intervention Programme (KiGIP) SLT trial. This was a randomised, controlled, parallel group, single masked clinical trial which tested the hypothesis that selective laser trabeculoplasty (SLT) is superior to timolol eye drops for the treatment of open-angle glaucoma, the design and main results have been previously reported.^13^

The KiGIP SLT trial was registered with the Pan African Clinical Trials Registry (PACTR201508001235339).

### Participants

Participants who attended the eye clinic at Kilimanjaro Christian Medical Centre, Moshi, Tanzania, were screened consecutively for eligibility between 31 August 2015 and 12 May 2017. Inclusion criteria for the trial were an IOP of >21 mm Hg, structural changes of the optic nerve head (DDLS score ≥5 or a vertical cup:disc ratio (vCDR) ≥0.7, or a vCDR asymmetry between two eyes of ≥0.2), and functional changes (glaucomatous VF defect, Mills GSS ≥1).^3^ Categories of high-risk glaucoma suspect (IOP >25 mm Hg, structural changes as previously mentioned and no VF defect) or high-risk ocular hypertension (IOP >32 mm Hg, no structural or functional defect) were also permitted. Exclusion criteria included participants being aged <18 years or eyes with no perception of light. More details are described elsewhere.^13^

### Diagnostic methods

The visual function was assessed using the logarithm of the minimum angle of resolution (logMAR) visual acuity measured at 2 m with the Peek Acuity Smartphone app V.3.5.0 (Peek Vision, London, UK) in a dimmed room.^14^ Static VF perimetry was performed using the Swedish interactive threshold algorithm standard 24–2 or 10–2 programs (II-I Series System software V.4.2, Humphrey HFA II 740i Visual Field Analyzer; Carl Zeiss Meditec AG, Jena, Germany).

Glaucoma-related structural features were assessed by slit-lamp examination of the anterior segment, pachymetry (central corneal thickness (CCT)), gonioscopy, fundus imaging and indirect fundoscopy (using a Digital 1.0X Volk slit-lamp lens) of the optic nerve head, macula and peripheral retina. The lens and the slit-lamp calliper were used to measure the optic nerve head diameter. The examiner of the enrolment visits was a single consultant ophthalmologist who followed a standard protocol and was masked to the VF examination, which was performed by a different investigator. All examinations were done prior to randomisation and treatment allocation.

The structural glaucomatous damage of the optic nerve head was classified using the DDLS, the vCDR and the horizontal CDR.^5 6^ DDLS was determined by locating the thinnest neuroretinal rim and, if still present, calculating the rim:disc ratio or, if absent, estimating the circumferential extension of the absence of neuroretinal rim tissue in degrees. After measuring the disc diameter, the DDLS was established accordingly. To determine the CDRs, the vertical and horizontal cup diameters were related to the respective disc diameters.

### Analysis

For the purpose of this post hoc analysis, all eyes enrolled in the trial were staged according to the mean deviation (MD) categories of Mills GSS including stage 5 (end-stage disease) if an eye was unable to perform a VF examination attributable to central scotoma.^3^ The functional GSS stages were used as the reference standard to compare with structural changes in advanced glaucoma.

The median GSS was used to subdivide eyes into two groups of glaucoma severity: (1) GSS 1–3 (early, moderate and advanced glaucoma) and (2) GSS 4–5 (severe and end-stage glaucoma). The performance of CDRs and DDLS for discriminating between these two groups of functional damage was evaluated with receiver operating characteristic (ROC) curves adjusted for intereye correlation. Curves were compared using the area under the receiver operating characteristic (AROC) curve. ROC curve analyses were also used to identify the best threshold to achieve the highest combination of specificity and sensitivity. In addition, AROCs were also calculated using the two groups GSS 1–2 (early, moderate) and GSS 3–5 (advanced, severe end-stage).

The correlation between the GSS, DDLS and the CDRs was assessed using Spearman’s rank correlation coefficient. The arithmetic mean CDR was calculated from the vCDR and horizontal CDR measurements.

Logistic regression models were constructed to determine potential risk factors of severe/end-stage glaucoma with generalised estimating equations adjusting for the correlation between eyes.^15^ The association between each potential risk factor and severe/end stage glaucoma was first estimated in a univariable (unadjusted) model before adjusting for confounding variables. Potential confounders were assessed through a change-in-estimate approach^16^ by adding covariates to the unadjusted model and retaining them if the OR of the covariate of interest changed by around 10% or more. Multicollinearity was checked for by evaluating change of SEs of the coefficient estimates.

## Results

A total of 201 participants (382 eyes) were enrolled in this study. Their mean age was 66.3 (SD 11.6) years and 83/201 (41%) were female (table 1). The VF assessments of participants’ eyes showed an average MD of −17.2 (SD 11.1) dB for 347 eyes using the 24–2 Humphrey VF test, and an average MD of −32.3 (SD 3.4) dB in eight eyes using the 10–2 test. A VF test was not possible for 27 eyes due to a low visual acuity (table 1).

**Table 1:** Patient and ocular characteristics

Patient characteristics (patients, n)	Total N=201
Sex, n (%)	
Female	83 (41.3)
Male	118 (58.7)
Age (years), mean (SD)	66.3 (11.6)
Education, n (%)	
<Secondary level	133 (66.2)
≥Secondary level	68 (33.8)
Ethnic group, n (%)	
Chagga	111 (55.2)
Pare	41 (20.4)
Meru	8 (4.0)
Maasai	5 (2.5)
Sambaa	5 (2.5)
Other	31 (15.4)
Financial resources (US$/day), n (%)	
≤2	76 (38.2)
>2	123 (61.8)
Travel distance (km), n (%)	
<50	105 (52.2)
≥50	96 (47.8)
Family history of glaucoma*, n (%)	
No	153 (76.1)
Yes	48 (23.9)

Ocular characteristics (eyes, n)	Total N=382
Prior topical glaucoma treatment, n (%)	
No	157 (41.1)
Yes	225 (58.9)
Prior timolol treatment, n (%)	
No	174 (45.5)
Yes	208 (54.5)
Pseudophakia, n (%)	
No	362 (94.8)
Yes	20 (5.2)
Exfoliation glaucoma, n (%)	
No	333 (87.2)
Yes	49 (12.8)
CCT (µm), mean (SD)†	521.0 (34.7)
Angle pigmentation (Spaeth), n (%)	
Light pigmentation (0–2)	320 (83.8)
Strong pigmentation (3–4)	62 (16.2)
Intraocular pressure (mm Hg), mean (SD)	26.7 (6.9)
Visual acuity, Snellen, WHO categories, ICD-11, n (%)	
No vision impairment (VA ≥6/12)	244 (63.9)
Mild vision impairment (6/18 ≤VA <6/12)	48 (12.6)
Moderate vision impairment (6/60 ≤VA <6/18)	40 (10.5)
Severe vision impairment (3/60 ≤VA <6/60)	3 (0.8)
Blindness (1/60 ≤VA <3/60)	2 (0.5)
Blindness (PL ≤VA <1/60)	44 (11.5)
Blindness (NPL)	1 (0.3)
Functional stage of glaucoma (GSS), n (%)	
Early	88 (23.0)
Moderate	55 (14.4)
Advanced	44 (11.5)
Severe	168 (44.0)
End stage	27 (7.1)
Disc Damage Likelihood Scale score, n (%)	
5	76 (19.9)
6	42 (11.0)
7	44 (11.5)
8	87 (22.8)
9	66 (17.3)
10	67 (17.5)
Disc Damage Likelihood Scale score (mean, SD)	8.0 (1.8)
VF, 24–2, MD (dB)‡, mean (SD)	−17.2 (11.1)
VF, 10–2, MD (dB)§, mean (SD)	−32.3 (3.4)

Data of 382 eyes at entry into the KiGIP SLT trial are mean (SD) or n (%).

*In a first-degree relative.

†CCT measurements missing in 13 eyes due to temporary failure of the pachymeter.

‡24–2 VF results of 347 eyes.

§10–2 VF results of eight eyes. No VF possible in 27 eyes due to reduced central vision.

CCT, central corneal thickness; GSS, glaucoma staging system; MD, mean deviation; VF, visual field.

**Figure 1:**
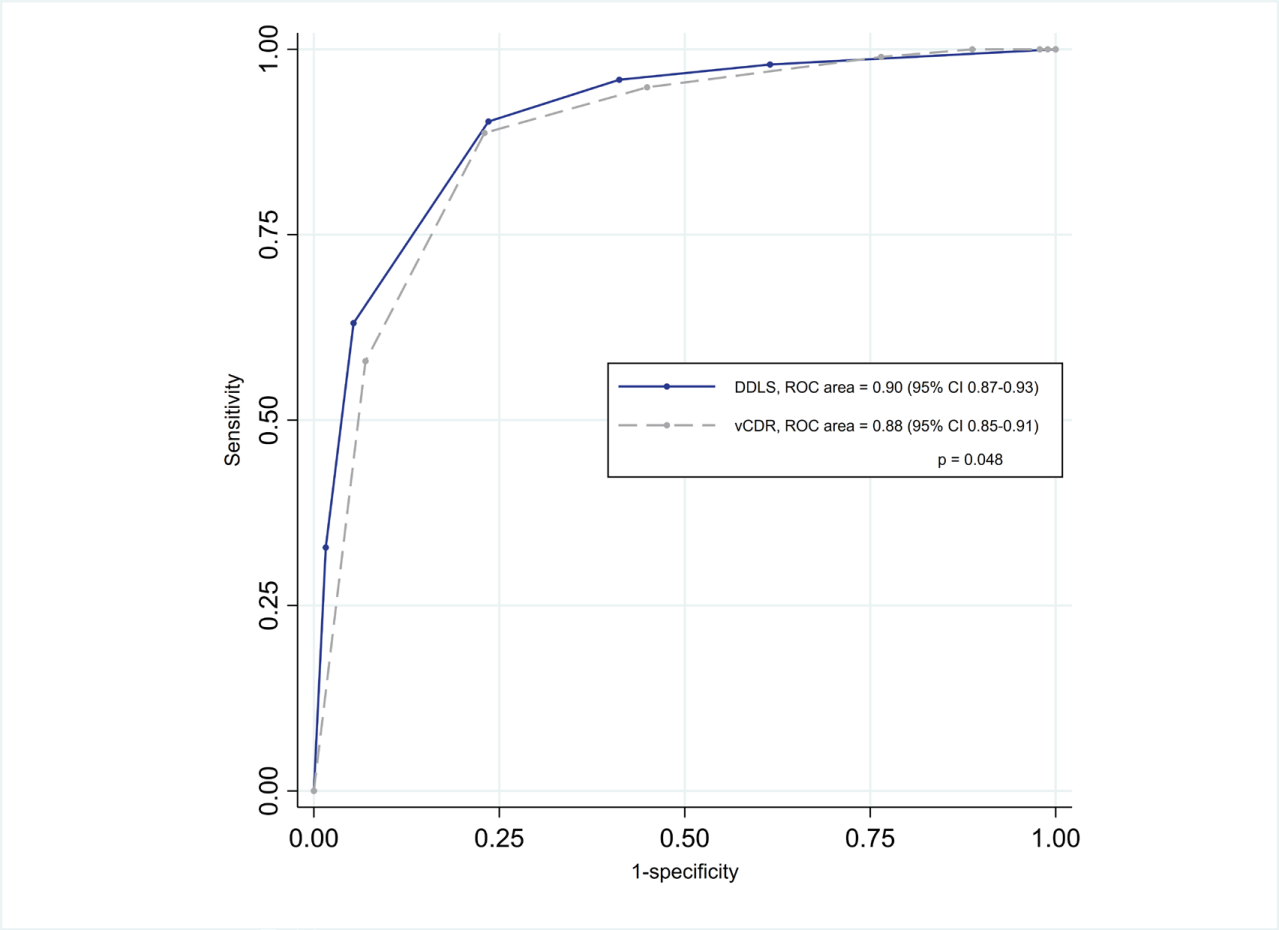
ROC curves of DDLS (solid line) and vCDR (dashed line) and the binary classifier of functional glaucoma stages: early/moderate/advanced versus severe/end stage. DDLS, Disc Damage Likelihood Scale; ROC, receiver operating characteristic; vCDR, vertical cup:disc ratio.

Subdividing the GSS into two groups following the median GSS resulted in 187 eyes (49.0%) with early/moderate/advanced glaucoma and 195 eyes (51.0%) with severe/end-stage glaucoma. Predicting this dichotomous variable using DDLS yielded an AROC curve of 0.90 (95% CI 0.87 to 0.93, figure 1). Using a cut-off point of DDLS score of 8 and above, 83.5% of eyes were correctly classified resulting in a sensitivity of 90.3% and a specificity of 76.5%. For the vCDR, the AROC curve was 0.88 (95% CI 0.85 to 0.91). Using a cut-off point of 0.9 and above, 83.0% of eyes were correctly classified with a sensitivity of 88.7% and a specificity of 77.0%. The difference in the two areas under the curve of DDLS and vCDR was statistically significant (p=0.04, figure 1). When combining the vCDR and horizontal CDR by calculating the mean CDR, the AROC curve was 0.89 (95% CI 0.86 to 0.93); sensitivity and specificity were 85.5% and 80.8%, respectively. Spearman’s rank correlation value comparing GSS with DDLS was 0.73 with vCDR of 0.71.

The alternative groups GSS 1–2 (early, moderate) contained 143 eyes (37%) and GSS 3–5 (advanced, severe, end-stage) 239 eyes (63%) resulting for DDLS in an AROC of 0.90 (95% CI 0.87 to 0.93) and for CDR of 0.90 (95% CI 0.87 to 0.93, p=0.64).

Risk factors associated with severe/end-stage glaucoma with a p value of <0.05 in univariable analyses were a lower level of education, less financial resources, presence of exfoliation glaucoma, lower CCT, higher IOP at baseline, presence of vision impairment and advanced structural stage of glaucoma (DDLS and vCDR). The adjusted analyses showed an association (p<0.05) between severe/end-stage glaucoma and financial resources of ≤US$2/day, IOP of ≥25 mm Hg, the presence of vision impairment (VA<6/12) and advanced structural stage of glaucoma (DDLS≥8, vCDR≥0.9; see also tables 2 and 3).

**Table 2:** Predicted ORs for functional severe/end-stage glaucoma at entry into the KiGIP SLT trial

Variable	Severe and end-stage glaucoma	Unadjusted analyses		Adjusted analyses	
	n/N (%)	OR (95% CI)	P value	OR (95% CI)	P value
Sex					
Female	77/159 (48)	1 (ref)		1 (ref)	
Male	118/223 (53)	1.18 (0.74 to 1.88)	0.49	0.96 (0.54 to 1.72)	0.90
Age groups (years)					
<70	118/233 (51)	1 (ref)		1 (ref)	
≥70	77/149 (52)	1.04 (0.65 to 1.67)	0.87	1.04 (0.65 to 1.67)	0.87
Education					
<Secondary level	139/251 (55)	1 (ref)		1 (ref)	
≥Secondary level	56/131 (43)	0.60 (0.37 to 0.98)	0.043	0.85 (0.43 to 1.67)	0.64
Ethnic group					
Chagga	99/209 (47)	1 (ref)		1 (ref)	
Pare	39/81 (48)	1.03 (0.57 to 1.85)		0.73 (0.34 to 1.59)	
Meru	12/15 (80)	4.59 (1.05 to 20.19)		2.63 (0.43 to 16.26)	
Maasai	5/9 (56)	1.49 (0.33 to 6.80)		1.09 (0.09 to 13.62)	
Sambaa	6/10 (60)	1.68 (0.38 to 7.40)		2.50 (0.34 to 18.44)	
Other	34/58 (59)	1.57 (0.81 to 3.07)	0.31*	1.18 (0.47 to 2.96)	0.70*
Financial resources (US$/day)					
≤2	91/143 (64)	1 (ref)		1 (ref)	
>2	104/239 (44)	0.44 (0.27 to 0.72)	0.00085	0.47 (0.24 to 0.95)	0.036
Travel distance (km)					
<50	100/201 (50)	1 (ref)		1 (ref)	
≥50	95/181 (52)	1.13 (0.71 to 1.79)	0.61	0.68 (0.35 to 1.33)	0.26
Family history of glaucoma†					
No	148/290 (51)	1 (ref)		1 (ref)	
Yes	47/92 (51)	1.01 (0.59 to 1.72)	0.98	1.55 (0.77 to 3.12)	0.22
Prior timolol treatment					
No	90/174 (52)	1 (ref)		1 (ref)	
Yes	105/208 (50)	0.95 (0.60 to 1.51)	0.84	1.15 (0.62 to 2.16)	0.66
Pseudophakia					
No	187/362 (52)	1 (ref)		1 (ref)	
Yes	8/20 (40)	0.79 (0.30 to 2.04)	0.62	0.39 (0.10 to 1.55)	0.18
Exfoliation glaucoma (XFG)					
No	165/333 (50)	1 (ref)		1 (ref)	
Yes	30/49 (61)	2.05 (1.03 to 4.09)	0.041	1.28 (0.47 to 3.45)	0.63
Central corneal thickness (µm)					
<520	106/181 (59)	1 (ref)		1 (ref)	
≥520	80/188 (43)	0.53 (0.34 to 0.84)	0.0064	0.70 (0.39 to 1.24)	0.22
Angle pigmentation					
Light pigmentation	164/320 (51)	1 (ref)		1 (ref)	
Strong pigmentation	31/62 (50)	0.90 (0.49 to 1.66)	0.74	1.61 (0.67 to 3.89)	0.29
Intraocular pressure (mm Hg)					
<25	57/175 (33)	1 (ref)		1 (ref)	
≥25	138/207 (67)	4.07 (2.59 to 6.41)	<0.0001	2.77 (1.54 to 4.97)	0.001
Vision impairment, VA <6/12					
No	88/244 (36)	1 (ref)		1 (ref)	
Yes	107/138 (78)	5.82 (3.68 to 9.22)	<0.0001	3.54 (1.89 to 6.64)	<0.001
Stage of glaucoma, DDLS					
Moderate (stages 5–7)	19/162 (12)	1 (ref)		1 (ref)	
Advanced (stages 8–10)	176/220 (80)	29.20 (16.19 to 52.64)	<0.0001	18.11 (9.59 to 34.20)	<0.001
Stage of glaucoma, vertical cup:disc ratio					
Moderate (<0.9)	22/166 (13)	1 (ref)		1 (ref)	
Advanced (≥0.9)	173/216 (80)	26.06 (14.67 to 46.30)	<0.0001	17.70 (9.40 to 33.34)	<0.001

Results of 382 eyes analysed at entry into the KiGIP SLT trial using unadjusted and adjusted logistic regression models with general estimating equations of potential factors associated with functional severity of glaucoma.

*Wald test for trend.

†In a first-degree relative.

DDLS, Disc Damage Likelihood Scale.

**Table 3:** DDLS

Glaucoma grade	DDLS stage	Definition	Anatomical descriptor
At risk	1	0.4≤RDr	Narrowest rim width (RDr)
	2	0.3≤RDr <0.4	
	3	0.2≤RDr <0.3	
	4	0.1≤RDr <0.2	
Glaucoma damage	5	RDr <0.1	
	6	1° ≤extension <45°)	Extent of rim absence (extension (°))
	7	45° ≤extension <90°	
Glaucoma disability	8	90° ≤extension <180°	
	9	180° ≤extension <270°	
	10	270° ≤extension	

The DDLS score is based on the narrowest radial neuroretinal rim width. As the rim width also depends on the disc size, the DDLS score should be increased by 1 for small discs (<1.50 mm) and decreased by 1 for large discs (>2.00 mm). Adapted from Spaeth *et al*.^6^

DDLS, Disc Damage Likelihood Scale; RDr, rim:disc ratio.

**Figure 2:**
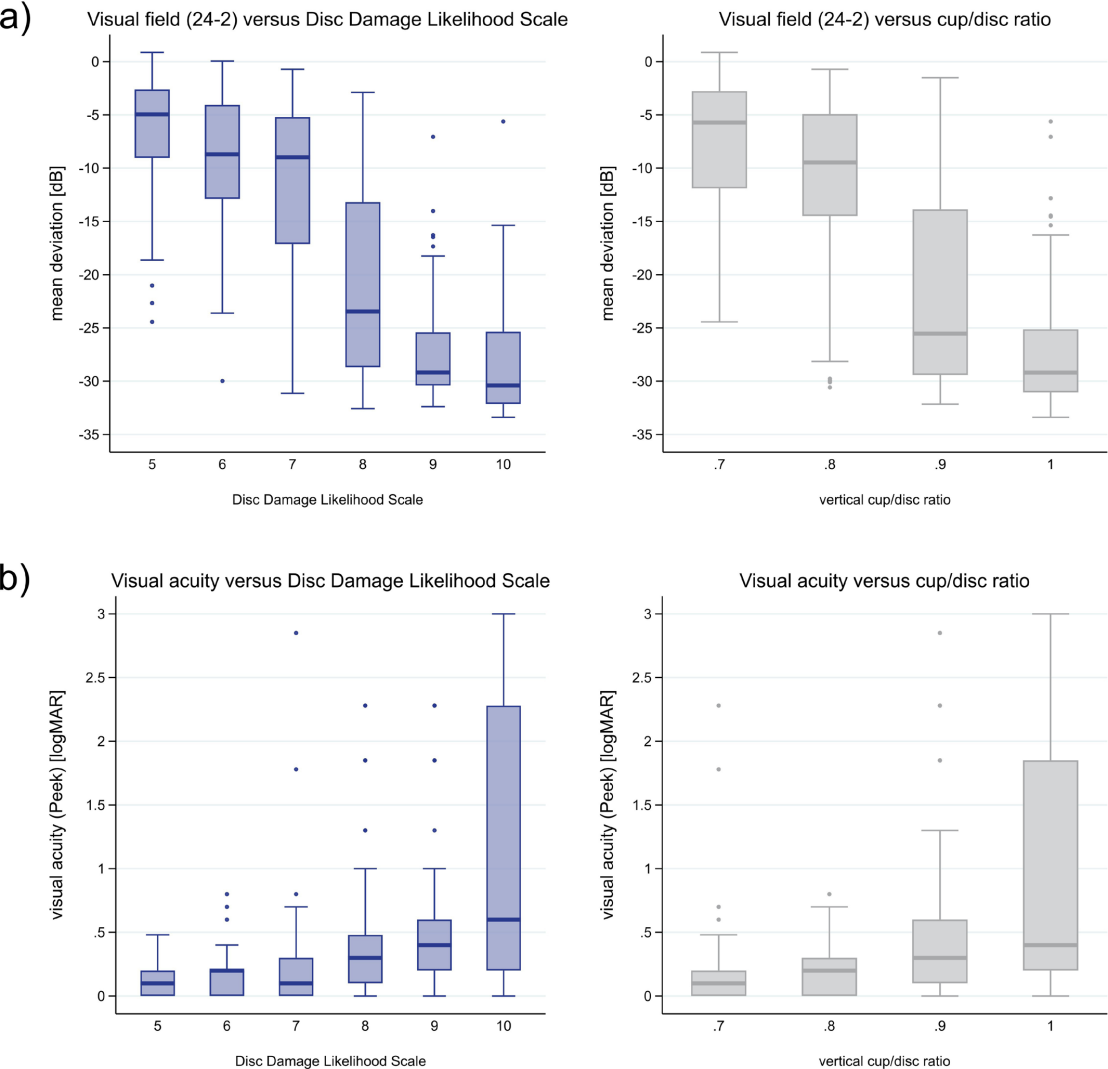
Comparison of the Disc Damage Likelihood Scale and vertical cup:disc ratio with (A) visual field mean deviation and (B) visual acuity (logMAR). Boxes show median, upper and lower quartiles. Whiskers represent scores outside the middle 50%. Outliers are presented as individual dots. logMAR, logarithm of the minimum angle of resolution.

Two functional descriptors of glaucomatous damage, static VF examination (continuous mean deviation) and visual acuity (logMAR) compared with two structural descriptors DDLS and vCDR are shown in figure 2. The mean deviation drops rapidly starting from DDLS score of 8 and vCDR of 0.9. Visual acuity initially increases slowly but shows a steep increase towards DDLS score of 10 and vCDR of 1.

## Discussion

This study found that the two structural optic disc staging systems, Armaly’s CDR^5^ and Spaeth *et al.*’s DDLS,^6^ were both able to discriminate between functionally mild/moderate/advanced glaucoma and severe/end-stage glaucoma. There was some evidence (p=0.048) of a larger AROC curve for DDLS compared with the vCDR.

Prior studies of DDLS have reported mainly AROCs for the discrimination between normal and glaucomatous eyes for the purpose of glaucoma detection. Danesh-Meyer *et al.* compared people without glaucoma and patients with glaucoma, defined by a combination of glaucomatous optic disc and VF changes and IOP. Clinical examination using DDLS had the highest AROC curve for identifying glaucoma from suspect or normal (AROC=0.91) followed by CDR (AROC=0.81), MD of VF examination (AROC=0.78), Hodapp-Parrish-Anderson VF score (AROC=0.75) and HRT-II rim area (AROC=0.62).^17^ Kara-José *et al.* similarly compared normal individuals to patients with early glaucoma and reported similar findings but with no significant differences between DDLS and CDR.^18^ Our results in more advanced glaucoma showed comparable AROCs which are noteworthy because optic disc changes are more pronounced in early glaucoma than in severe or end-stage disease compared with functional tests.^7 19^ The ocular hypertension treatment study showed that the earliest signs of progression from ocular hypertension to glaucoma are more likely detected by structural changes of the optic disc than by functional VF changes.^20^ The results from this study suggest that DDLS and, to a lesser extent, vCDR can provide a staging of the glaucomatous optic disc damage up to end-stage glaucoma, including stages where automated perimetry is no longer possible. Then the optic disc grading may be supplemented by visual acuity measurements. The visual acuity categories ‘hand movement’ and ‘counting fingers’ are separated by three 0.1 logMAR units or ‘lines’ at 30 cm confirming the clinical impression that the difference is relevant for a person with severe or end-stage glaucoma even beyond the possibility of using a static VF device.^9^

AROCs of CDRs increased slightly in our study when using the mean of vCDRs and horizontal CDRs. A possible explanation for this might be that early glaucomatous changes of the neuroretinal rim thickness start in the inferotemporal and superotemporal parts of the cup,^21^ predominantly captured by the vCDR. Temporal and lastly nasal neuroretinal rim areas are affected as the glaucomatous damage progresses to more advanced stages, increasingly captured by the horizontal CDR as well.^22^ This typical course of thinning of the different sectors of the optic disc rim is also reflected in the two anatomical descriptors of the DDLS (narrowest rim thickness (DDLS scores 1–5) and extension of rim absence (DDLS scores 6–10, table 3). DDLS therefore also allows finer grading of advanced glaucoma stages compared with CDR (figure 2), which is only based on changes of the cup diameter.

Rim:disc ratio also performed better than CDR in fully automated fundus image processing to categorise optic discs when comparing them to expert clinician annotations.^23^ DDLS has been shown to be more accurate and repeatable than the CDR^24 25^ and is also used in community screening and shared glaucoma care models in New Zealand and Scotland, for example.^26 27^

Apart from the anatomical descriptors DDLS and CDR, the current study found a higher IOP was a risk factor for severe and end-stage glaucoma, which has previously been reported by several other studies including from Africa.^28 29^ Financial resources of ≤US$2/day of a patient were another risk factor for severe and end-stage glaucoma. Several studies report associations between advanced glaucoma and a low socioeconomic status.^30 31^ This is also in line with a general link between poverty and an increased risk of vision impairment.^1 30 31^

Our study has several limitations. The data were acquired during a clinical trial by examiners who followed standard operating procedures, but the data were not externally validated with image analysis. We were also not able to capture consistent optical coherence tomography (OCT) images: after a failure of the initially used time-domain OCT device, it had to be replaced by a spectral-domain OCT device, whose measurements were not interchangeable.^32^ While OCT can be useful in assessing advanced glaucoma,^33^ described limitations included artefacts and segmentation errors, and the OCT reference database may not be relevant for the particular patient.^34^ A further limitation of the current study is that trial participants were randomised to treatments rather than glaucoma severity, which could bias the results of the post hoc analysis. Furthermore, two exposures of interest and the outcome were involved in the inclusion criteria for the trial (cut-offs of DDLS score ≥5 or CDR ≥0.8, GSS >0). This could mean that the estimates of the strength and size of the association between each of these and the outcome could be different in a more general population which also includes all patients with glaucoma. Furthermore, measurements of functional and structural parameters are subject to multifactorial variability (eg, physiological, examiner-related, eye-related and device-related), which must always be taken into account. These variations were also present in our dataset, for example, as outliers (see figure 2).

In conclusion, DDLS and CDR are low-cost and feasible methods for describing and discriminating structural stages related to functionally mild/moderate/advanced glaucoma versus severe/end-stage glaucoma. The DDLS may be advantageous over the CDR due to the slightly larger AROC, more categories to differentiate advanced glaucoma and a better fitting description of the course of glaucomatous optic disc damage. This study supports the use of these grading systems also in advanced glaucoma. They can be implemented with affordable equipment without a need for complex technology and when a VF examination cannot be performed, for example, in patients with severe or end-stage glaucoma or a strong fatigue effect or in young children. Both can play an important role in the assessment of advanced glaucoma damage and progression and help clinicians with treatment decisions to prevent further visual disability.

## Data Availability

Data are available upon reasonable request.
